# The Edmonton Obesity Staging System for Pediatrics (EOSS-P) in Mexican Children and Adolescents Living with Obesity: Beyond BMI Obesity Classes

**DOI:** 10.3390/children12111556

**Published:** 2025-11-17

**Authors:** Isabel Omaña-Guzmán, Roberto Carlos Rodríguez Quintero, Arturo Ruíz-Arroyo, Edith Prado Díaz, Juan Carlos López-Alvarenga, Ana María Hernández López, Zendy Fuentes Corona, Karina Aguilar Cuarto, Karen Pedraza Escudero, Alejandra Ruíz Barranco, Erendira Villanueva-Ortega, Nayely Garibay-Nieto

**Affiliations:** 1 Pediatric Obesity Clinic and Wellness Unit, Hospital General de México “Dr. Eduardo Liceaga”, Mexico City 06720, Mexico; nutricion.isa@gmail.com (I.O.-G.); rc.roberto.sb@hotmail.com (R.C.R.Q.); ruizaj22@gmail.com (A.R.-A.); pradoedith3@gmail.com (E.P.D.); anahello7@gmail.com (A.M.H.L.); zendy_ff@outlook.es (Z.F.C.); kari.cuarto@gmail.com (K.A.C.); karenpedrazaescudero@outlook.es (K.P.E.); aleruba30@gmail.com (A.R.B.); villorte@yahoo.com (E.V.-O.); 2 Division of Population Health & Biostatistics, School of Medicine, University of Texas Rio Grande Valley, Edinburg, TX 78539, USA; juan.lopezalvarenga@utrgv.edu

**Keywords:** pediatric obesity, classification, Mexico, EOSS-P

## Abstract

**Highlights:**

**What are the main findings?**
  • More than half of the children were classified in stage 3, the most severe stage of the EOSS-P.
  • There is a weak association between EOSS-P staging and BMI-based obesity classification in children.
**What is the implication of the main finding?**
  • The assessment of obesity in children should go beyond BMI and include metabolic, mechanical, mental, and social domains
  • The EOSS-P is a valuable tool for the comprehensive assessment of obesity that could be adapted to different contexts.

**Abstract:**

**Background/Objectives:** The Edmonton Obesity Staging System (EOSS) was developed to stage the obesity in adult populations. Subsequently, this staging system was designed for pediatric populations (EOSS-P). This study aimed to describe obesity severity using EOSS-P and correlate it with BMI classes in Mexican children and adolescents living with obesity. **Methods:** This is a cross-sectional analysis carried out with data from school-age children and adolescents living with obesity who were referred to the Pediatric Obesity Clinic at the Child Welfare Unit at the General Hospital of Mexico “Dr. Eduardo Liceaga”. Obesity was staged using the EOSS-P. To evaluate the association between obesity classes and each EOSS-P domain, as well as overall EOSS-P staging, we performed Bayesian ordered logistic regression models. **Results:** A total of 118 participants were included; 43.2% were female and 56.8% were male. Based on the overall EOSS-P staging, 56.8% of participants were classified as stage 3, while none were categorized as stage 0. Obesity class II-III was associated with higher odds for the mechanical (OR = 2.5), metabolic (OR = 1.9), and social (OR = 1.6) domains. **Conclusions:** Pediatric obesity assessment should extend beyond BMI to include the evaluation of metabolic, mechanical, and psychological domains, identifying health complications and barriers that may impact treatment effectiveness and adherence. The EOSS-P is a valuable tool for staging pediatric obesity based on these domains and can guide personalized clinical decision-making.

## 1. Introduction

Obesity is a chronic disease characterized by an excess of adipose tissue [1]. This condition represents a significant public health issue globally. According to the World Obesity Federation, it is projected that, by 2030, 254 million children and adolescents aged 5–19 will be living with obesity [2]. Mexico, like other countries, is currently facing a major pediatric health concern, as projections estimate that by 2030, nearly 254 million children and adolescents aged 5–19 will be living with obesity worldwide. Over the past four decades, the rise in obesity prevalence has been almost twice as high in the Americas compared to the rest of the world, with Mexico showing the most pronounced increase within the region [3].

This disproportionate increase has led to health expenditures attributable to overweight accounting for 8.9% of total health spending, while the overall economic impact has reached about 5.3% of GDP, representing the largest burden worldwide [4]. Despite the government’s efforts to reduce this problem, obesity has increased in recent decades. According to the National Survey of Health and Nutrition (ENSANUT), in 2022, the national prevalence of obesity in children (5 to 11 years) was 18.1%, while in adolescents it was 17.1% [5].

The etiology and maintenance of obesity involve the interaction of biological, socioeconomic, and environmental factors [6]. Obesity in children and adolescents is associated with short- and long-term complications such as hypertension, dyslipidemia, insulin resistance, metabolic syndrome, polycystic ovary syndrome, sleep disorders, alterations in posture, depression, and anxiety, among others [6,7].

The Body Mass Index (BMI) percentile for age and sex is a recommended tool for diagnosing and classifying obesity in the pediatric population. BMI does not directly measure body fat mas [8]. However, its use along with age in children and adolescents has shown a good correlation with the fat mass index and lean mass index, but a lower correlation with fat mass percentage [9]. Despite its limitations, the BMI is a valuable and practical tool in clinical practice for screening and diagnosing obesity [8]. Nevertheless, obesity is a complex disease that requires a comprehensive assessment beyond the BMI since multiple organs, systems, and social domains are affected [10]. Considering the above, the Edmonton Obesity Staging System (EOSS) was developed to stage obesity in adult populations based on the presence of comorbidities and functional limitations [11]. Subsequently, a similar system was designed for pediatric populations (EOSS-P). The EOSS-P included the assessment of comorbidities and social issues affecting the pediatric population in each domain [12].

EOSS-P included four evaluation domains related to the presence of obesity that can affect the therapeutic management: (1) metabolic, (2) mechanical, (3) mental health and (4) social milieu. Each domain is classified according to the severity, and the global staging is based on the most severely affected domain. This system aims to provide health professionals with more information to address effective therapeutic strategies [12].

In adult populations, EOSS stratification has been related to a higher risk of all-cause mortality [13,14] and adequately predicts adverse outcomes in patients with COVID-19 [15]. In children, there are few studies that have evaluated the use of EOSS-P. In children from Canada [16], Greece [17], and Australia [18], it was observed that BMI obesity classes do not consistently correlate with EOSS-P stages. In Canadian adolescents, higher stages of EOSS-P were related to a decrease in quality of life [19].

This study aimed to describe the obesity severity using EOSS-P and its association with obesity BMI classes in Mexican children and adolescents living with obesity.

## 2. Materials and Methods

### 2.1. Study Design and Population

This is a retrospective and cross-sectional study that included data from children and adolescents referred to the Pediatric Obesity Clinic at the Child Welfare Unit at the General Hospital of Mexico “Dr. Eduardo Liceaga” between August 2023 and August 2024. This institution is a high-specialty public hospital that primarily serves a population with low to lower-middle socioeconomic status. The majority of these individuals do not have social security and have limited incomes, making the hospital a crucial center for providing care to vulnerable populations in Mexico City.

The Pediatric Obesity Clinic offers multidisciplinary and family-centered care for children and adolescents who are living with obesity. Most of the patients are referred from primary and secondary care units. Since 2023, the EOSS-P system was implemented as part of clinical care to assess the stage of obesity.

A non-probabilistic convenience sampling method was employed, based on feasibility considerations and accessibility to the target population. For this analysis, data recorded in the medical records of patients who had undergone EOSS-P evaluation were used. The inclusion criteria were: (1) Ages 7 to 17 years, (2) A diagnosis of obesity, and (3) First-time admissions to the Pediatric Obesity Clinic with no history of prior intervention for obesity. Cases of endogenous, endocrine, or genetic obesity were excluded.

This protocol was approved by the Committee for Research Projects of Medical Residents, with Registration Number DECS/JPO-CT-1449-2022, at Hospital General de México “Dr. Eduardo Liceaga”.

### 2.2. Obesity Diagnosis

Weight and height were measured with a Digital measuring station SECA 284. The diagnosis of obesity was performed using the CDC references for BMI percentile for age and sex [20]. Obesity classes were defined according to the percentage of BMI above the 95th percentile: class 1 obesity at a BMI of ≥100% of the 95th percentile, class 2 obesity at a BMI of ≥120% of the 95th percentile, and class 3 obesity at a BMI of ≥140% of the 95th percentile.

### 2.3. EOSS-P

**Figure 1:**
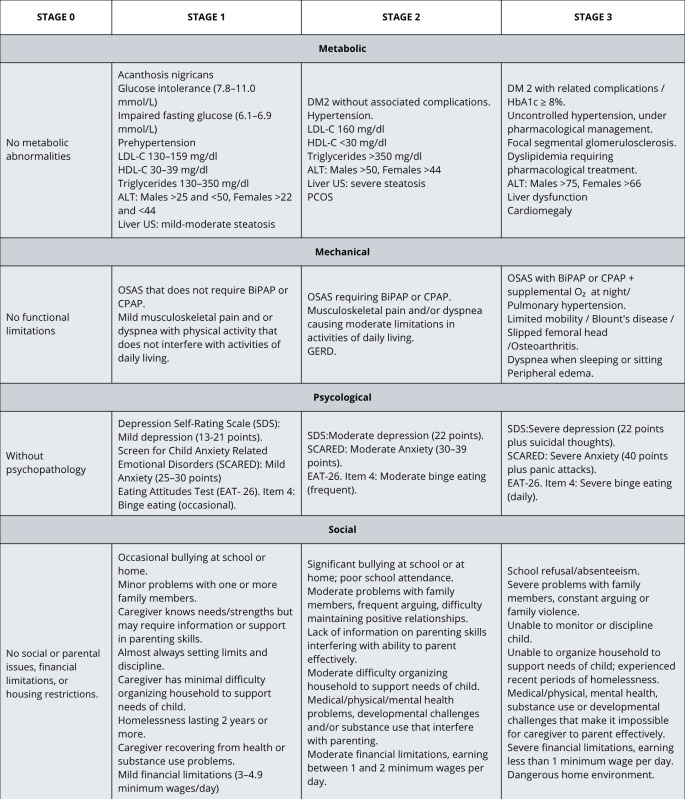
EOSS-P adapted to Mexican children. Abbreviations: ALT, alanine transaminase; CPAP, continuous positive airway pressure; DM2, type 2 diabetes mellitus; GERD, gastroesophageal reflux disease; HDL-C, high-density lipoprotein cholesterol; LDL-C, low-density lipoprotein cholesterol; OSAS, obstructive sleep apnea syndrome; PCOS, polycystic ovary syndrome.

The EOSS-P was used to stage the obesity. Figure 1 illustrates the characteristics evaluated in each domain and their severity at each stage.

Metabolic domain. This domain was evaluated based on the following indicators:

- Acanthosis nigricans. A pediatrician determined the presence of acanthosis on the neck, armpits, and groin and used the classification proposed by Burke and cols [21].
- Blood pressure (BP). BP was measured in a sitting position, after a 5 min rest, and with an arm support so that the antecubital fossa was at the level of the heart. The sphygmomanometer was long enough to cover 80–100% of the arm circumference at the midpoint between the olecranon and the acromion and had a width equivalent to 2/3 of the circumference. Two measurements were taken during the consultation, and the average of these was obtained. To identify the presence of prehypertension or hypertension, BP was classified according to percentiles [22].
- Biochemical data. Fasting serum concentrations of glucose, insulin, total cholesterol, HDL cholesterol, LDL cholesterol, triglycerides, and alanine aminotransferase (ALT) were determined by standardized methods. When fasting glucose was >100 mg/dL, an oral glucose tolerance test was performed to identify Type 2 diabetes mellitus or prediabetes.
- Liver steatosis. Liver ultrasound was requested for all patients.
- Polycystic Ovary Syndrome (PCOS). PCOS was diagnosed in females according to the Rotterdam criteria [23], requiring the presence of at least two of the following: clinical or biochemical hyperandrogenism, oligo/anovulation, or polycystic ovarian morphology on ultrasound. Hirsutism was assessed using the Ferriman–Gallwey scoring system for women [24], which evaluates nine androgen-sensitive body areas, each scored from 0 to 4. A total score of ≥8 was considered indicative of generalized hirsutism.

Mechanical domain. This domain was evaluated by a sports physician using targeted examination and validated tools. This domain comprises the assessment of obstructive sleep apnea syndrome (OSAS), which was evaluated using a reduced version of the Pediatric Sleep Questionnaire developed to assess sleep disorders in children [25]. A score < 0.33 was considered the absence of OSAS. A sports physician assessed musculoskeletal pain and complications limiting physical activity through clinical examination. Gastroesophageal reflux disease (GERD) was assessed based on the presence of at least one of the following symptoms occurring two or more times per week: chronic heartburn, regurgitation, chest or retrosternal pain (after meals, awakens patients from sleep, feeling of oppression or burning, radiates to the back, lasts minutes or hours, exacerbated by stress), nausea, dysphagia and epigastric pain. Finally, dyspnea was evaluated using the New York Heart Association (NYHA)/Ross modification classification [26].

Mental Health domain. This domain was evaluated by a psychologist using the Depression Self-Rating Scale [27], the Screen for Child Anxiety-Related Emotional Disorders (SCARED) [28], and one question from the Eating Attitudes Test (EAT-26) [29] regarding the presence of binge eating.

- Depression Self-Rating Scale. This scale evaluates moderate to severe depression in childhood. The total score of the scale was classified as follows: (1) without psychopathology: 0–12 points; (2) mild depression: 13–21 points; (3) moderate depression: 22 points; (4) severe depression: 22 points plus suicidal thoughts. These cut-off points were proposed in a study conducted among Peruvian children to validate a modified version of the Depression Self-Rating Scale [30].
- Screen for Child Anxiety Related Emotional Disorders (SCARED). This self-report instrument was developed to screen anxiety disorders in children [28]. The total score was categorized as follows: without psychopathology (0–24 points), mild anxiety (25–30 points), moderate anxiety (30–39 points), and severe anxiety (40 points plus panic attacks).
- Eating Attitudes Test (EAT-26). EAT-26 is a standardized self-report tool to measure symptoms and characteristics of eating disorders [29]. To evaluate the presence and frequency of binge, we considered item four of this scale (Have gone on eating binges where I felt that I may not be able to stop).

Social domain. The social domain was evaluated with a questionnaire designed by a social worker specifically for the EOSS-P evaluation (Supplementary Material S1). Based on the questionnaire results, the stages were defined as shown in Figure 1.

### 2.4. Statistical Analysis

A descriptive analysis was performed to characterize the studied population. Means and standard deviations (SD) were estimated for continuous variables and frequencies and proportions for categorical variables. To identify differences between obesity classes and sex, we employed chi-squared and Fisher’s exact tests.

To evaluate the association between obesity classes and each EOSS-P domain, as well as overall EOSS-P staging, we performed Bayesian ordered logistic regression models. We considered a Bayesian analysis due to the small sample size. The Bayesian approach estimated Odds ratios for ordered logistic regression and its 95% of credible interval. We used 12,500 Markov Chain and Monte Carlo iterations with 2500 initial burn-in (these were used to warm up the iterations). The obtained efficiency was between 0.03 and 0.04 with acceptance rate between 0.21 and 0.24. The calculated Monte Carlo Standard Error was smaller than standard deviation means, suggesting stable estimates.

The appropriateness of the model was tested on the proportional odds assumption using generalized ordered logit models. In every case, the Wald test did not reject the proportional odd assumption (all *p*-values *p* > 0.05). All models were adjusted by sex and age group (school-age children aged 6–11 years and adolescents aged 12–17 years). We combined the obesity class II and III groups to obtain aggregated estimation of the coefficients because of the small sample size of the class III group. Before merging these groups, we confirmed that there were no significant differences in age or EOSS-P staging between them.

We also fitted parallel frequentist ordered logistic models to generate predicted probabilities for each EOSS-P stage by obesity class, adjusted for age and sex. These predictions are presented descriptively and were consistent with the Bayesian model estimates.

We presented estimated *p*-values, those <0.05 were considered significant. All analyses were performed in Stata version 15.0 (Stata Corp., College Station, TX, USA).

## 3. Results

### 3.1. Participant Characteristics

A total of 118 participants were included, of whom 43.2% were female and 56.8% male (Table 1). The mean age was 11.4 years (SD = 2.5). Overall, 56% were school-aged children (6–11 years), and 44% were adolescents (12–18 years). The majority were classified as having class 1 obesity (64.4%). No significant differences in obesity class distribution were found between females and males. Additionally, no statistical differences in the age of obesity onset were identified between the obesity class groups [6.5 (2.9) years vs. 5.8 (3.5) years, *p* = 0.255].

### 3.2. EOSS-P Staging

According to the metabolic domain of the EOSS-P Scale, 79.9% of children were classified in stage 1, 13.6% in stage 2, and 5.9% in stage 3 (Table 2). In stage 1, the most common alterations were acanthosis nigricans, present in 91.3% of children, followed by hypertriglyceridemia (55.8%), low HDL-C (48.6%), and moderate liver steatosis (29.5%). In stage 2, elevated ALT levels (≥50 U/L in males; ≥44 U/L in females) were the most prevalent alteration, affecting 40% of the studied group, followed by hypertension, observed in 25% of cases. In stage 3, the most common alteration was elevated ALT levels (≥75 U/L in males; ≥66 U/L in females) (85.7%).

In the mechanical domain, over half of the children were classified in stage 1 (54.2%), with none in stage 3. In stage 1, OSAS was the most common alteration, affecting 72.2% of children. In stage 2, all children were diagnosed with GERD with no other alterations present.

Regarding the psychological domain, most of the children were classified in stage 1 (31.4%) and stage 0 (28%). In those with stage 1, the most prevalent alterations were mild anxiety (45.9%) and mild depression (43.2%). In stages 2 and 3, moderate anxiety (95%) and severe anxiety (75%) were the most common psychopathologies.

As for the social domain, it was alarming that most children were found in stages 2 (56.8%) and 3 (40.7%). In all stages, the primary issues were financial limitations, which varied by grade and stage. Additionally, in stage 2.7% of parents were found to lack sufficient information about parenting skills.

Regarding the overall EOSS-P stage, most of the participants were classified into stage 3 (56.8%). Notably, no participants were assigned to stage 0.

No significant differences were observed between children living with obesity class I and those with obesity class II/III in any EOSS-P domain or in the overall staging.

Across all four EOSS-P domains, the distribution of scores was broadly similar between obesity class I and class II–III, with substantial overlap across categories (Supplementary Figure S1). This even dispersion of responses supports the challenges of detecting differences using frequentist approaches and the application of Bayesian ordered logistic regression to stabilize parameter estimation in this small sample.

### 3.3. Association Between Obesity Classes and EOSS-P Staging

**Figure 2:**
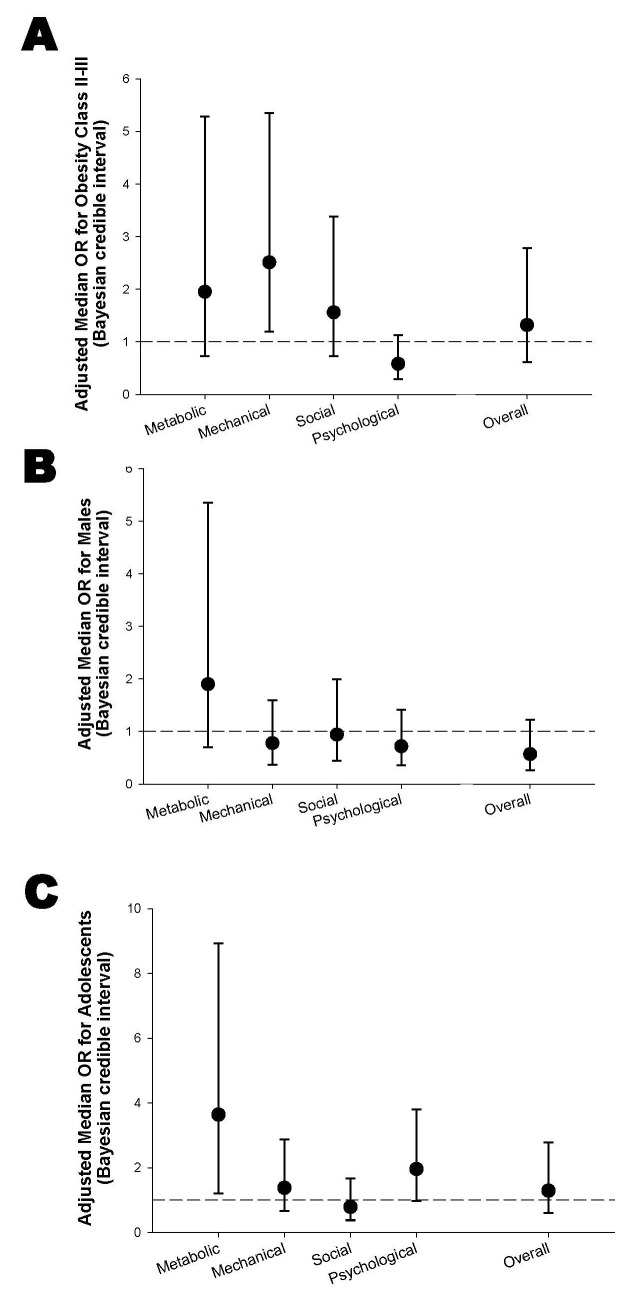
Adjusted odds ratios (ORs) with 95% Bayesian credible intervals for predictors of EOSS-P domains. (**A**) ORs for obesity class II/III versus obesity class I, (**B**) ORs for males versus females, and (**C**) ORs for adolescents (12–17 years) versus school-age children (6–11 years). Outcomes include the four EOSS-P domains (Metabolic, Mechanical, Social, Psychological) and the overall EOSS stage. The dashed horizontal line at OR = 1 represents the null value.

The Bayesian ordered logistic regression models showed distinct patterns across predictors (Figure 2). Obesity class II–III, compared with class I, was associated with higher odds across metabolic (OR = 1.9, 95% credible interval: 0.732, 5.291), mechanical (OR = 2.5, 95% credible interval: 1.194, 5.352), social (OR = 1.6, 95% credible interval: 0.734, 3.376), and overall EOSS staging (OR = 1.3, 95% credible interval: 0.607, 2.949), while the psychological domain showed a point estimate below 1 (OR = 0.6, 95% credible interval: 0.287, 1.136) (Figure 2A). Males, compared with females, displayed modestly increased odds for metabolic (OR = 2.2, 95% credible interval: 0.701, 5.352) but near-null or attenuated effects across other domains, with overall EOSS showing ORs below unity (Figure 2B). Adolescents, compared with school-age children, demonstrated the largest point estimates, particularly for the metabolic domain (OR = 3.2, 95% credible interval: 1.214, 8.929) (Figure 2C).

Overall, these results suggest that obesity class and age group have a mild influence on the higher burden of the EOSS-P compared to sex. However, most of the estimates were accompanied by wide Bayesian credible intervals, some overlapping the null value, consistent with the limited sample size.

**Figure 3:**
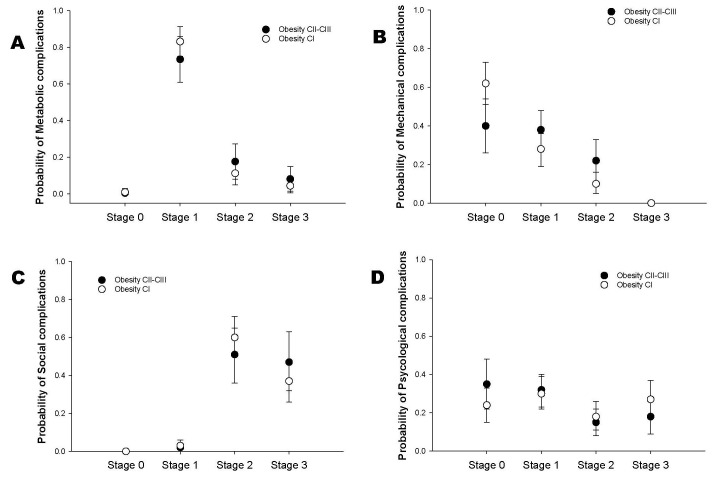
Predicted probabilities of EOSS-P stages by obesity class. Probabilities were estimated from ordered logistic regression models adjusted for age and sex. The panels correspond to: (**A**) metabolic, (**B**) mechanical, (**C**) social, and (**D**) psychological complications. Circles represent predicted probabilities for obesity class I (open) and obesity classes II–III (solid), with vertical bars indicating 95% confidence intervals. The figure illustrates that metabolic complications showed higher probabilities for obesity class II–III at intermediate EOSS stages, whereas other domains exhibited largely overlapping distributions across obesity classes.

Predicted probabilities from the ordered logistic models (Figure 3) showed that children and adolescents with obesity class II–III had higher probabilities of metabolic complications at EOSS stages 1 and 2 compared with those in class I, whereas probabilities for stages 0 and 3 were similar (Figure 3A). For mechanical, social, and psychological domains, predicted probabilities overlapped considerably between obesity classes, indicating no clear separation in risk profiles (Figure 3B–D). These patterns were consistent with the Bayesian regression findings, although the Bayesian framework was used for inference.

## 4. Discussion

Our findings underscore the importance of conducting comprehensive assessments in children and adolescents living with obesity, as metabolic, mechanical, and psychological alterations—together with social barriers—are present across all BMI categories. We found evidence of an association between BMI categories and EOSS-P staging. However, the Bayesian credible intervals approached the null value, indicating uncertainty for some of the domains. This uncertainty may partly reflect the inherent variability of the EOSS-P structure, in which all domains contribute equally to the overall stage, and a single severe finding can elevate the classification. While this feature increases clinical sensitivity, it may also reduce predictive precision when used as a composite outcome. Nevertheless, our application of Bayesian modeling provides a contextually relevant framework for evaluating how EOSS-P captures multidimensional complications beyond BMI alone.

*Metabolic domain*. Other studies have found a positive association between obesity classes and the metabolic domain when age and sex were considered as confounders. For example, in children from Canada [16] and Australia [18], those with obesity class III had a higher risk of having a stage 2 or 3 rating in the metabolic domain. In contrast, in our sample, obesity classes showed only a mild association with metabolic alterations after adjustment for sex and age group.

In our sample, obesity class I was the most prevalent (64.4%), whereas class III was least frequent (7.6%). In contrast, the prevalence of class III was clearly higher in Australian (24.2%) and Canadian (27%) studies [16,18]. Despite the lower prevalence of severe obesity in our sample, metabolic complications were common across all BMI categories. Only 1.3% of children with class I obesity were classified in metabolic stage 0, compared with over 20% in Canadian children [16] and 12% in Australian children [18]. Interestingly, none of the Mexican children with class II/III obesity were classified in stage 0. These findings support a higher burden of metabolic alterations in Mexican children, reinforcing the need for direct metablic assesment.

The differences outlined above may stem from genetic and environmental factors that increase the risk of metabolic alterations in Mexican children. Previous studies have shown that Mexican children [31] and Hispanic adolescents [32] exhibit a higher prevalence of metabolic abnormalities compared to non-Hispanic White and non-Hispanic Black populations. Furthermore, Mexican young adults have been documented to possess a genetic predisposition to insulin resistance (IR), metabolic syndrome, and obesity [33]. This genetic susceptibility could partly explain our findings: nine out of ten children in metabolic stage 1 presented with acanthosis nigricans, a clinical marker of IR. Low HDL-C was the third most common metabolic alteration observed, consistent with findings in Mexican adults, among whom low HDL-C is the most prevalent form of dyslipidemia, strongly linked to genetic factors [34].

Adolescents with obesity exhibit a higher proportion of metabolic complications than preadolescents, likely due to the physiological surge in insulin resistance during puberty [35], progressive adiposity accumulation, chronic inflammation, oxidative stress, and redistribution of visceral fat, which exacerbates dyslipidemia, insulin resistance, and fatty liver risk [36]. Lifestyle factors common in adolescence such as food insecurity, reduced physical activity, and disrupted sleep, can increase metabolic vulnerability. In our study, adolescents supported association with metabolic alterations, consistent with these mechanisms.

*Mechanical domain*. The mechanical domain was the least affected overall, with stages 0 and 1 varying across obesity classes, as expected. Compared with Australian children [18], Mexican children had a lower prevalence of stage 0 and higher prevalence of stages 1 and 2. No participants were classified as stage 3, similar to the very low prevalence rates reported in Australia [18]. As in Canadian [16] and Australian [18] cohorts, obesity class II/III increased the risk of mechanical stage 2 or 3, confirming this domain was clearly associated with BMI categories. This association likely reflects the direct mechanical burden of excess of adiposity on respiratory and locomotor systems. The most frequent mechanical alteration was obstructive sleep apnoea syndrome (OSAS), which impairs neurocognitive development, academic performance and cardiovascular health [37,38].

*Psychological domain*. Our findings are consistent with the Australian study [18], with no association between obesity class and psychological stage. However, fewer Mexican children were in stage 0 (28% vs. 55.4%), suggesting a greater burden of mental health issues. Studies in Mexican [39] and other populations [40] have similarly reported higher rates of depression and anxiety among youth with obesity, though stratification by obesity class remains limited.

Binge eating without compensatory behaviors was rare in our sample; most affected children (77%) also exhibited compensatory behaviors such as vomiting or food restriction. Given that 63% of participants experienced some degree of binge eating, assessing eating risk behaviors as an integrated construct may be more informative than evaluating binge eating alone.

*Social domain*. In the social domain, none of the children were classified as stage 0 and only a few as stage 1, indicating that most families faced moderate to severe social barriers likely affecting treatment adherence. Financial hardship was the most frequent issue, consistent with the socioeconomic profile of patients attending the General Hospital of Mexico “Dr. Eduardo Liceaga,” which primarily serves individuals without social security coverage. Although a meta-analysis [41] reported that higher socioeconomic status increases the risk of obesity among Mexican youth, vulnerable populations remain underassessed despite facing substantial barriers that hinder care. Social environment factors—such as family practices, household conditions, and school settings—have been linked to obesity risk across studies in the United States and Latin America [42], while low socioeconomic status and poor mental health are predictors of non-adherence to treatment [43].

The questionnaire used to assess the social domain was tailored to the sociodemographic characteristics of our clinic population, which differs markedly from the Canadian context where the EOSS-P was developed. This instrument is currently being validated by our group. Developing culturally adapted tools is essential to ensure accurate social assessment and improve the clinical applicability of the EOSS-P across diverse populations.

*Factors linked to explanation of differences among studies*. The differences observed between our results and those from other countries may be explained by several factors. Genetic and epigenetic backgrounds, particularly affecting the metabolic domain, likely contribute, as discussed previously. Socioeconomic disparities also play a central role: while Canada and Australia are high-income countries, Mexico is classified as upper-middle-income, with lower public health expenditures and more limited healthcare coverage [44]. Consequently, Mexican children experience restricted access to medical care and healthy foods, alongside greater exposure to food insecurity and ultra-processed diets, all of which heighten metabolic risk.

Pediatric outcomes should not be framed as adult endpoints such as mortality or cardiovascular events but rather as functional and developmental trajectories. Current tools, including the EOSS-P, remain underdeveloped for capturing these trajectories within quantitative frameworks. Social determinants of health—poverty, food insecurity, stigma, and inadequate access to care—strongly influence both metabolic and psychological outcomes. Low socioeconomic status predicts higher obesity prevalence, while stigma and bullying mediate depression and anxiety; neighborhood and family environments further shape these risks. Although the overlap of these domains is conceptually recognized in the EOSS-P, it remains poorly represented in empirical models.

We should consider that the EOSS-P treats all complications as equally important, even though they carry very different risks and costs. A single abnormal finding is treated the same as multiple abnormalities. The variability observed in our predicted density analysis can be explained by the construction of the index, which inherently reflects this nature. Additionally, by design, the highest score in any domain determines the overall stage. This means that a single moderate abnormality can elevate a child’s stage, even if other domains are normal.

To address these inconsistencies, weighting domain-specific components may improve EOSS-P performance by better stratifying children according to treatment needs and resource allocation. Recent approaches in adults propose domain weighting methods that could be adapted for pediatric populations [45]. The EOSS framework continues to evolve and should integrate BMI with relevant health determinants to reflect the social and clinical realities of each geographic region [46]

*Study limitations*. A limitation of this study is the non-probabilistic convenience sampling method, which restricts the generalizability of results to other populations and introduces selection bias due to the clinical and sociodemographic characteristics specific to the pediatric population served at the Hospital. Moreover, the small number of children with class III obesity also limited our ability to assess the gradients of alterations across EOSS-P domains. Therefore, it is necessary to perform studies in other clinical and social contexts with representative samples. Otherwise, binge eating was evaluated using a single item from a broader scale not specifically designed for this behavior, which may have reduced measurement accuracy. Nonetheless, this approach revealed that disordered eating behaviors, beyond binge eating, are prevalent among children and adolescents attending our clinic. Future studies in Mexico should incorporate validated instruments for assessing a wider range of eating disorders.

To our knowledge, this is the first study in a Mexican pediatric population to examine the association between obesity classes and EOSS-P staging. A key strength lies in the evaluation being conducted by an experienced multidisciplinary team. Furthermore, the social domain questionnaire was developed specifically for the population served by the Pediatric Obesity Clinic of the Child Welfare Unit and designed by an expert social worker with extensive field experience in our institution.

*Implications for clinical practice*. Our findings emphasize the limitations of BMI as a sole indicator of obesity severity and support the adoption of comprehensive staging systems such as EOSS-P, which integrate metabolic, mechanical, psychological, and social dimensions. The recent inclusion of EOSS-P in Mexico’s National Medical Care Program (PRONAM) represents an important step toward early identification of comorbidities and individualized treatment at the primary care level. Monitoring its implementation will be essential to evaluate impact and refine practice guidelines.

Beyond clinical care, the use of EOSS-P can help policymakers identify priority needs and allocate resources to improve both the physical and mental health of children while addressing the obesogenic environments in which they live. Recognizing obesity as a chronic, non-communicable disease that begins in childhood underscores the urgency of investing in early prevention and specialized care. Such investments can yield substantial long-term benefits by reducing the future burden of metabolic and cardiovascular diseases.

## 5. Conclusions

In conclusion, although BMI remains a widely used diagnostic and classification tool for obesity, it is a weak predictor of complications in children, as it does not account for body composition, fat distribution, or metabolic status. Integrating BMI with the EOSS-P framework enhances clinical assessment by incorporating metabolic, mechanical, psychological, and social dimensions. In pediatric populations, outcomes should emphasize the risk of reaching young adulthood with these complications rather than adult endpoints such as mortality. Social determinants—poverty, stigma, and limited access to care—strongly influence psychological distress and health behaviors, underscoring the need for tools tailored to the specific sociocultural context of the population studied. Longitudinal studies are warranted to evaluate the prognostic value of EOSS-P in Mexican children and adolescents.

The severity of obesity was most consistently associated with a higher probability of metabolic complications, particularly at intermediate EOSS stages. In contrast, mechanical, social, and psychological complications overlapped across obesity classes, suggesting that factors beyond BMI drive their progression. Adolescence emerged as a stronger determinant of overall EOSS-P burden than sex, highlighting the importance of the developmental stage in risk stratification. Collectively, these findings support the use of EOSS-P to capture the heterogeneity of obesity-related complications and demonstrate the utility of Bayesian modeling for inference in small pediatric samples.

## Figures and Tables

**Table 1:** Participant characteristics and EOSS-P staging.

	Total n = 118 Mean (SD)	Females n = 51 Mean (SD)	Males n = 67 Mean (SD)	*p*-Value
**Age (years)**	11.4 (2.5)	11.9 (2.4)	11.0 (2.4)	0.057
**Height (cm)**	150.5 (12.7)	151.2 (12.1)	150.0 (13.6)	0.620
**BMI Z-score**	2.1 (0.32)	2.1 (0.25)	2.1 (0.37)	0.444
**Obesity diagnosis**	**n (%)**	**n (%)**	**n (% )**	***p*-value**
Obesity Class I	76 (64.4)	37 (72.5)	39 (58.2)	0.205
Obesity Class II	33 (28.0)	10 (19.6)	23 (34.3)	
Obesity Class III	9 (7.6)	4 (7.8)	5 (7.5)	
**Group age**	**n (%)**	**n (%)**	**n (%)**	***p*-value**
School age children	66 (56%)	24 (47.1)	42 (62.7)	0.090
Adolescents	52 (44%)	27 (52.9)	25 (37.3)	

Data compared by chi-squared or Fisher’s exact tests, and Student’s *t*-tests.

**Table 2:** EOSS-P staging in participants.

EOSS Domains	Total n = 118 n (%)	Obesity Class I n = 76 n (%)	Obesity Class II/III n = 42 n (%)	*p*-Value
**Metabolic domain stage**				
0	1 (0.8)	1 (1.2)	0 (0.0)	0.489
1	94 (79.7)	63 (82.9)	31 (73.8)	
2	16 (13.6)	8 (10.5)	8 (19.1)	
3	7 (5.9)	4 (5.3)	3 (7.1)	
**Mechanical domain stage**				
0	64 (54.2)	47 (61.9)	17 (40.5)	0.065
1	37 (31.4)	21 (27.6)	16 (38.1)	
2	17 (14.4)	8 (10.5)	9 (21.4)	
3	0			
**Psychological domain stage**				
0	33 (28.0)	19 (25.0)	14 (33.3)	0.318
1	37 (31.4)	22 (28.9)	15 (35.7)	
2	20 (16.9)	13 (17.1)	7 (16.7)	
3	28 (23.7)	22 (28.9)	6 (14.3)	
**Social domain stage**				
0	0	0	0	0.541
1	3 (2.5)	2 (2.6)	1 (2.4)	
2	67 (56.8)	46 (60.5)	21 (50.0)	
3	48 (40.7)	28 (36.8)	20 (47.6)	
**Overall EOSS-P stage**				
0	0	0	0	0.796
1	2 (1.7)	1 (1.3)	1 (2.4)	
2	49 (41.5)	33 (43.4)	16 (38.1)	
3	67 (56.8)	42 (55.3)	25 (59.5)	

Data compared by chi-squared and Fisher’s exact tests.

## Data Availability

The data presented in this study are available on request from the corresponding author. The data are not publicly available due to the mandatory confidentiality as data belongs to pediatric patients of a Public Institution.

## Supplementary Materials

The following supporting information can be downloaded at https://www.mdpi.com/article/10.3390/children12111556/s1: Table S1: Social domain EOSS-P. Figure S1: Distribution of EOSS-P staging among obesity groups in each domain.

## Abbreviations

The following abbreviations are used in this manuscript:

BP	Blood Pressure
BMI	Body Mass Index
EOSS-P	Edmonton Obesity Staging System—Pediatric
PCOS	Polycystic Ovary Syndrome
ALT	Alanine transaminase
CPAP	Continuous positive airway pressure
DM2	type 2 diabetes mellitus
GERD	gastroesophageal reflux disease
HDL-C	high-density lipoprotein cholesterol
LDL-C	low-density lipoprotein cholesterol
OSAS	obstructive sleep apnea syndrome
SD	Standard deviation
