# Supplementary material for: The Edmonton Obesity Staging System for Pediatrics (EOSS-P) in Mexican Children and Adolescents Living with Obesity: Beyond BMI Obesity Classes
Source: Children (Basel). 2025 Nov 17;12(11):1556. doi: 10.3390/children12111556 (PMC12651275; doi:10.3390/children12111556)
Supplement: Supplementary file 1 [file children-12-01556-s001.zip › Supplementary Table S1.pdf]

EDMONTON OBESITY STAGING SYSTEM -PEDIATRIC (EOSS-P)

Social domain

Patient's name:____

ECU: ____________________ **Family type:** ____________________________**Date:** ____________________

Main economic provider: _________________________________ **Medical diagnosis:**_________________

0. Who within the family unit is the primary caregiver of the patient?

Both parents	One parent	Grandparents	Others – Who?

1. Has the patient expressed to you, or have you observed any type of bullying at school?

a) Physical and/or verbal bullying
b) Cyberbullying
c) Emotional bullying

No issues	Emphasis on the form of aggression (a)	Emphasis on the medium (b)	Emphasis on the object of aggression (c)

1.1. Have you observed any form of abuse of the patient within the family unit/home?

No abuse	Physical abuse	Psychological abuse	Neglect	Sexual abuse

2. Have you observed any difficulties in the patient's relationships with family members?

* Minor problems: Occasional arguments with a family member.
* Moderate problems: Situations that limit interaction among family members.
* Severe problems: Frequent arguments or domestic violence with parents, siblings, and/or other family members.

No issues	Minor problems	Moderate problems	Severe problems

3. Does the primary caregiver understand the patient's basic needs (yes ☐ / no ☐) and strengths (yes ☐ / no ☐) within the family context?

* Basic needs: Acceptance, love, respect, food, rest, playtime, freedom of expression, medical care.
* Strengths: Honesty, reliability, protectiveness, kindness, industriousness, independence, solidarity, etc.

Yes	Aware of needs, but requires support	Lacks information and requires parenting guidance	Incapable of providing care

3.1. When behavioral limits and rules are set for the patient, are they effective?

Always	Almost always	Sometimes	Incapable of monitoring and disciplining

4. Does the primary caregiver have difficulty organizing the household and meeting the patient's needs?

No difficulties – Functional support network	Minimal difficulty – Functional support network	Moderate difficulty – Unstable support network	Incapable

4.1. What is the family's housing situation?

Own	Rented	Borrowed	No housing and/or institutionalized

5. Is there any physical/medical, mental health, or substance use issue affecting the primary caregiver that limits their ability to care for the patient?

No limitations	In recovery	Interferes with parenting	Incapable of effective parenting

Reason: ____

6. Are there any financial limitations?

No limitations - Socioeconomic level 6	Mild limitations - Socioeconomic level 4–5	Moderate limitations - Socioeconomic level 2–3	Severe limitations - Socioeconomic level 1

7. Is there any legal situation and/or a dangerous domestic environment?

☐ Yes ☐ No Why? ____

☐ First time ☐ Recurrence

EOSS Social Environment: ____________________
