# Supplementary figures and images for: The Edmonton Obesity Staging System for Pediatrics (EOSS-P) in Mexican Children and Adolescents Living with Obesity: Beyond BMI Obesity Classes
Source: Children (Basel). 2025 Nov 17;12(11):1556. doi: 10.3390/children12111556 (PMC12651275; doi:10.3390/children12111556)

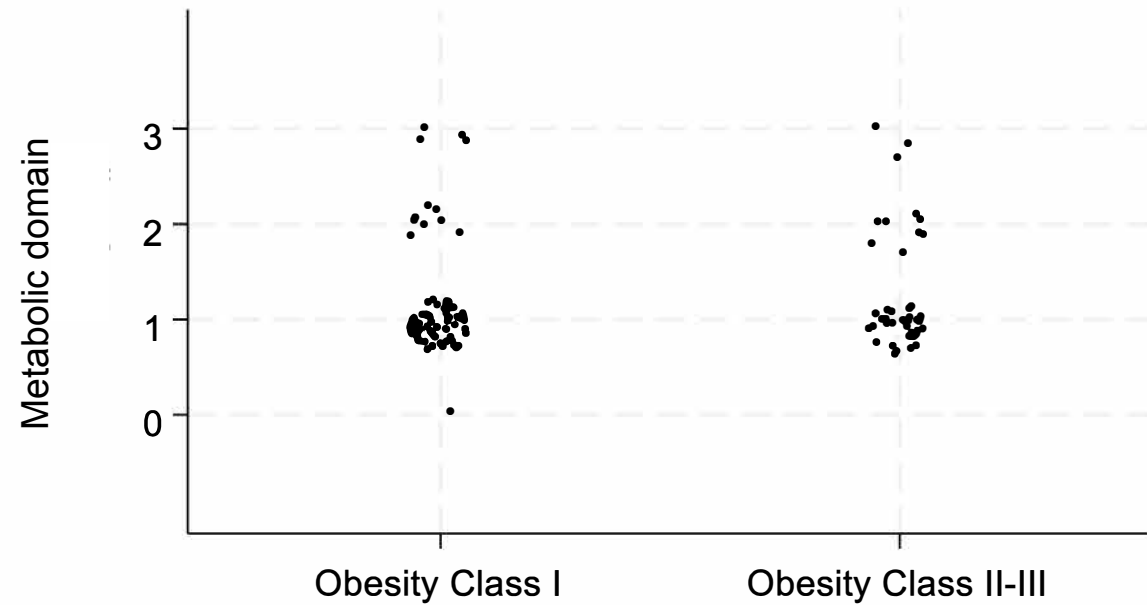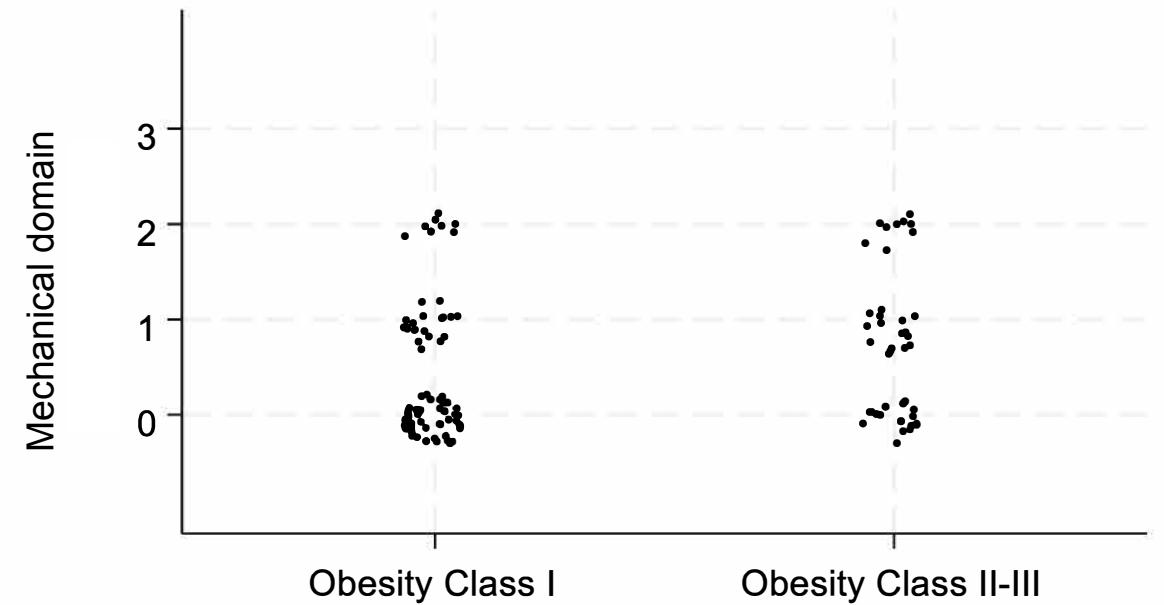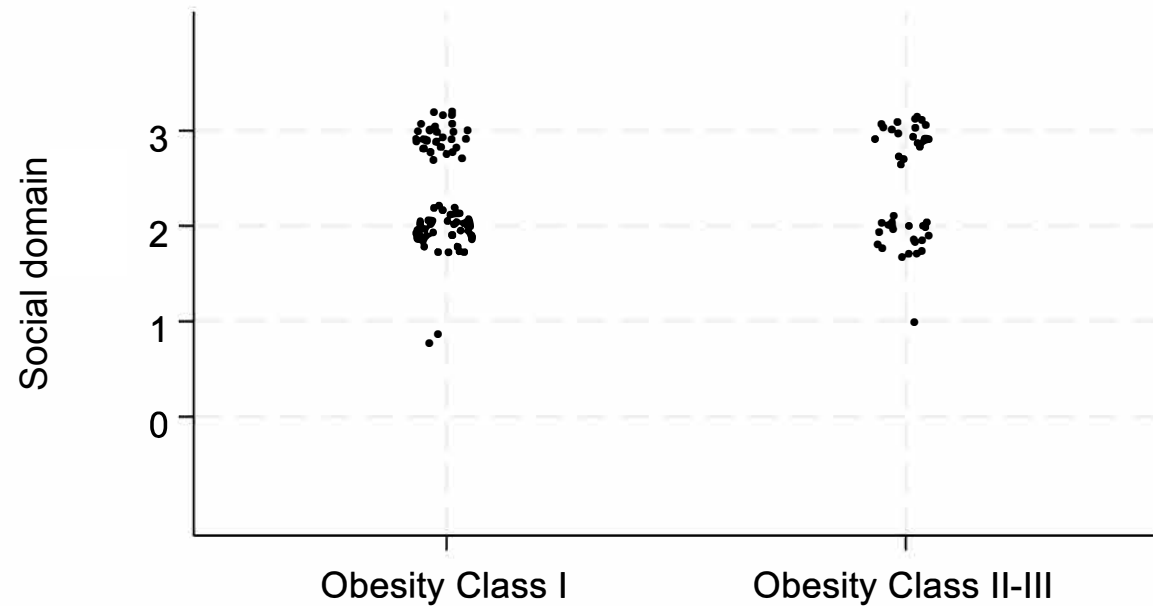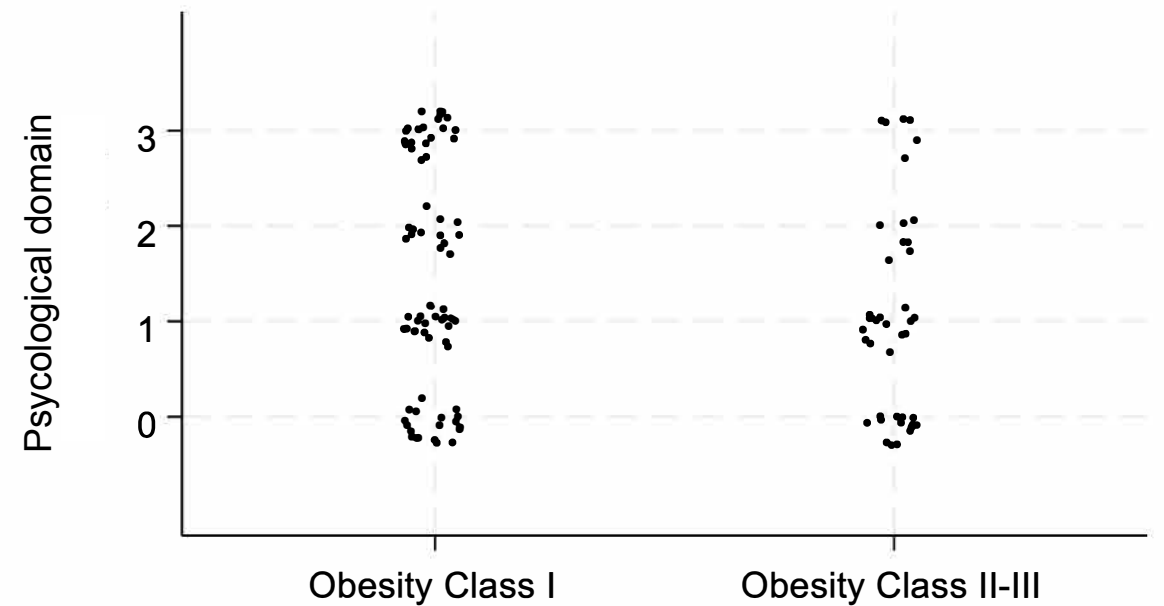

Supplement: Supplementary file 1 [file children-12-01556-s001.zip › Supplementay Figure S1.pdf]
